# Distinct Characteristics of Mandibular Bone Collagen Relative to Long Bone Collagen: Relevance to Clinical Dentistry

**DOI:** 10.1155/2014/769414

**Published:** 2014-04-10

**Authors:** Takashi Matsuura, Kentaro Tokutomi, Michiko Sasaki, Michitsuna Katafuchi, Emiri Mizumachi, Hironobu Sato

**Affiliations:** Section of Fixed Prosthodontics, Department of Oral Rehabilitation, Fukuoka Dental College, 15-1 Tamura 2-Chome, Sawara-ku, Fukuoka 814-0193, Japan

## Abstract

Bone undergoes constant remodeling throughout life. The cellular and biochemical mechanisms of bone remodeling vary in a region-specific manner. There are a number of notable differences between the mandible and long bones, including developmental origin, osteogenic potential of mesenchymal stem cells, and the rate of bone turnover. Collagen, the most abundant matrix protein in bone, is responsible for determining the relative strength of particular bones. Posttranslational modifications of collagen, such as intermolecular crosslinking and lysine hydroxylation, are the most essential determinants of bone strength, although the amount of collagen is also important. In comparison to long bones, the mandible has greater collagen content, a lower amount of mature crosslinks, and a lower extent of lysine hydroxylation. The great abundance of immature crosslinks in mandibular collagen suggests that there is a lower rate of cross-link maturation. This means that mandibular collagen is relatively immature and thus more readily undergoes degradation and turnover. The greater rate of remodeling in mandibular collagen likely renders more flexibility to the bone and leaves it more suited to constant exercise. As reviewed here, it is important in clinical dentistry to understand the distinctive features of the bones of the jaw.

## 1. Introduction

Bone is a dynamic tissue that undergoes constant remodeling in order to maintain a healthy skeleton. In clinical dentistry, jawbones frequently require surgical procedures, such as extraction of teeth, periodontal surgery, and implant surgery with or without bone regeneration. Of the many regenerative experiments for bone, few have been tested in the jaw. Because of the unique properties of the jawbone tissue, dentists and dental researchers should be aware that the data regarding other skeletal bones may not be entirely applicable to jawbones.

It is well recognized that the jawbone is remodeled faster than the other skeletal bones [1]. Jaw development is similar to that in other craniofacial bones but distinct from the axial and appendicular skeleton. The jaw arises from neural crest cells of the neuroectoderm germ layer rather than the mesoderm [2] and undergoes intramembranous, instead of endochondral, ossification [3]. Skeletal diseases such as cherubism [4], hyperparathyroid jaw tumor syndrome [5], and bisphosphonate-related osteonecrosis [6] occur only in the jaw. In case of ovariectomy and malnutrition, it is reported that the rat mandible loses trabecular bone and mineral density at a lower rate than the tibiae do [7]. Mesenchymal stem cells or bone marrow stromal cells derived from the jaw show higher osteogenic potential and additional distinctive features compared to other skeletal bones [8–12].

These distinctions owe partly to the unique characteristics of the jawbone matrix. It is important both for regenerative dental surgery and for maintenance of teeth or implants thereafter that dentists and dental researchers are knowledgeable of the unique features of the jawbone matrix. Though there are many bone matrix components, this review focuses on collagen, the most abundant matrix protein in bone and a determinant of bone strength and quality [13]. Collagen biochemistry is not well characterized in the maxilla; therefore, we focus on research findings for the mandible. We will first describe the role of collagen in bone matrix organization. We will then compare the characteristics of mandibular collagen to long bones to highlight the unique properties of the jawbone matrix that are relevant to clinical dentistry.

## 2. Role of Collagen on Bone Matrix Organization

Bone matrix consists mainly of a two-phase composite material: mineral and fibrillar collagen. Type I collagen comprises approximately 95% of the entire collagen content of bone. The other types of collagen, such as types III [16] and V [17], are at low levels and appear to modulate the diameter of type I collagen fibrils [17]. Mineral and fibrillar type I collagen are closely associated with each other; the latter functions as a three-dimensional template that organizes the former's deposition and growth [18]. Bone acquires its durability against external forces through this well-organized architectural arrangement between mineral and type I collagen fibrils.

The nature and extent of posttranslational modifications of collagen, many of which are unique to collagen [19], are related to the organization of mineral and collagen fibrils [18]. One such modification, the intermolecular, covalent crosslinking of collagen initiated by the enzymatic oxidative deamination of specific lysine (Lys) and hydroxylysine (Hyl) residues by lysyl oxidase (LOX), contributes to bone strength. In fact, the inhibition of LOX activity by lathyrogens impairs crosslinking, which leads to decreased bone strength caused by increased solubility and abnormal structure of collagen fibrils [20, 21].

Another modification, enzymatic hydroxylation of specific Lys residues by lysyl hydroxylase (LH), also can control bone matrix organization. The Hyl serves as a site of glycosylation [22, 23], and the resultant glycosylated residues affect collagen maturation [23–25], fibrillogenesis, and mineralization [22, 23]. In addition, this modification determines the pattern of intermolecular crosslinking of collagen. Among the 3 isoforms of LH (LH1, 2, and 3), LH2b, a spliced variant of LH2, catalyzes the hydroxylation of Lys residues in the C- or N-terminal, nontriple helical domain (i.e., the telopeptide domain) of collagen, which then directs the subsequent crosslinking towards the hydroxylysine (Hyl^ald^) pathway in mineralized tissues specifically [26]. Ectopic activation of the Hyl^ald^ pathway by overexpression of LH2b leads to defective collagen fibrillogenesis and matrix mineralization [27]. LH1 catalyzes Lys hydroxylation in the triple helical domain (helical domain), while LH3 has LH activity and, more importantly, galactosylhydroxylysine glucosyltransferase activity [22].

**Figure 1:**
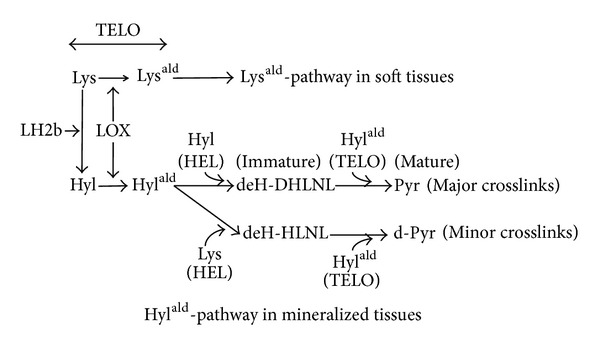
The collagen cross-linking pathway in mineralized tissues. The collagen cross-linking pathway in soft tissues arises from the Lys^ald^ both at the C- and N-telopeptide domains (Lys^ald^-pathway). In mineralized tissue, it does so from the Hyl^ald^ mostly at the C-telopeptide domain (Hyl^ald^-pathway). In mineralized tissues, the Lys residue at the C-telopeptide domain is converted into Hyl through the action of LH2b, followed by the conversion of the Hyl into the Hyl^ald^ through the action of LOX. To make the major crosslinks, the immature crosslink, deH-DHLNL, is first formed by pairing of the Hyl^ald^ with the Hyl at the helical domain of the neighboring molecule (Hyl^ald^  × Hyl), and the mature crosslink, Pyr, is then formed by a spontaneous condensation reaction (Hyl^ald^  ×  Hyl^ald^  ×  Hyl). To make the minor crosslinks, the immature crosslink, deH-HLNL, is formed (Hyl^ald^  ×  Lys), and then the mature crosslink, d-Pyr, is formed (Hyl^ald^  ×  Hyl^ald^  ×  Lys). The value of Pyr/deH-DHLNL presents collagen maturation rate. Lys^ald^: the aldehyde form of Lys; Hyl^ald^: the aldehyde form of Hyl; TELO: the telopeptide domain; HEL: the helical domain.

**Figure 2:**
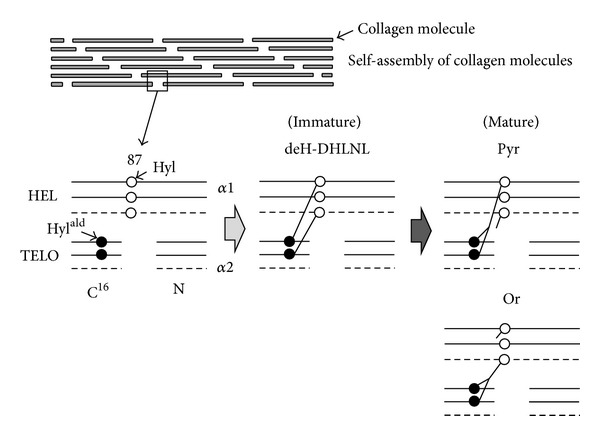
Diagram of the formation and maturation of the major collagen crosslink in mineralized tissues. The procollagen molecule secreted from the cell is processed by cleavages of both the N- and C-terminal propeptide extensions. The processed collagen molecules then self-assemble through clusters of charge and hydrophobicity of the triple helical domain of the molecule to form a fibril. The molecules in the fibril are then stabilized by extensive intermolecular crosslinking. The crosslink involves the Hyl^ald^ at the 16^C^ residue on the C-telopeptide domain of the two *α*1 chains and the Hyl at the 87 residue on the triple helical domain of the two *α*1 chains or the single *α*1 and *α*2 chains. The immature crosslink, deH-DHLNL, is formed by pairing of the Hyl^ald^ with the Hyl of the neighboring molecule. The mature crosslink, Pyr, is then formed; it owes its origin to the two Hyl^ald^ of *α*1 chains and the one Hyl of *α*1 or *α*2 chain. If the 87th residue on the helical domain is Lys, the minor immature crosslink, deH-HLNL, is formed, and then the minor immature crosslink, d-Pyr, is made up. The solid and dotted lines represent *α*1 and *α*2 chains, respectively. Hyl^ald^: the aldehyde form of Hyl; HEL: the helical domain; TELO: the telopeptide domain; N: N-terminal telopeptide domain; C^16^: the 16^C^ residue on the C-telopeptide domain; 87: the 87th residue on the helical domain.

At the beginning of the bone-specific cross-linking pathway, the Hyl residue in the telopeptide domain (formed by LH2b) is converted into an aldehyde (Hyl^ald^) by LOX (Figure 1). The iminium divalent intermolecular crosslinks are the first to form and they then mature into trivalent crosslinks through condensation reactions (Figure 2). The pairing of Hyl^ald^ with Hyl (formed by LH1) in the helical domain of the neighboring molecule forms the iminium crosslink, dehydrodihydroxylysinonorleucine (deH-DHLNL). By contrast, when the Hyl^ald^ pairs with Lys, dehydrohydroxylysinonorleucine (deH-HLNL) is formed. The major mature crosslink, pyridinoline (Pyr), is a maturational product of deH-DHLNL, formed by any of the following condensation reactions: (1) condensation of their two keto-amines through elimination of a Hyl [28], (2) condensation of a keto-amine and a Hyl^ald^ [29], or (3) condensation of a deH-DHLNL and its keto-amine [30]. A minor, mature cross-link form is deoxypyridinoline (d-Pyr), a lysyl analog of Pyr, made up of two Hyl^ald^ and one Lys in the helical domain [31].

Because these cross-link condensation reactions are usually spontaneous, turnover rate is an important factor in regulating cross-link maturation. For instance, periodontal ligament collagen has low levels of mature cross-links due to its high rate of turnover and, in turn, is more readily degraded due to lack of stable cross-links [25]. As for bone collagen, the levels of the major mature cross-link form, Pyr, versus the major immature cross-link form, deH-DHLNL, indicate the collagen maturation rate [32]. More recently, the research group of Yamauchi has demonstrated that the degree of Hyl glycosylation also influences cross-link maturation [23].

The galactosylhydroxylysine glucosyltransferase activity of LH3 promotes the formation of glucosylgalactosyl (GG)-Hyl from galactosyl (G)-Hyl at the cross-linking site. G- or G free-deH-DHLNL can mature into G-Pyr or G free-Pyr, while GG-deH-DHLNL cannot mature into GG-Pyr. Suppression of galactosylhydroxylysine glucosyltransferase activity of LH3 decreases the speed of cross-link maturation, reduces the amount of both immature and mature crosslinks, increases the diameter of collagen fibrils, and impairs matrix mineralization.

Two articles have demonstrated disordered bone collagen in LOX or LH knock-out mice. Pischon et al. [33] reported that LOX knock-out mice showed a perinatal lethality and that the craniofacial bone of the fetus at embryonic day 18.5 exhibited fragility and thinner collagen fibrils. Their osteoblast cultures revealed retard of osteoblastic differentiation and matrix mineralization. Takaluoma et al. [34] documented that LH1 knock-out mice were viable and fertile but 15% of them were led to sudden death mainly due to aortic ruptures. The femoral bone collagen of the adult LH1 knock-out mice showed a 75% and a 47% smaller amount of Hyl and the major mature crosslink, Pyr, respectively, compared to those of the adult wild-type mice. By contrast, the amount of a minor mature crosslink, d-Pyr, was 1284% greater, and thereby, total amount of Pyr and d-Pyr became 195% greater. Though collagen fibrils and matrix mineralization were not investigated, LH1 deficiency probably affects bone collagen matrix. As described above, the posttranslational modifications of collagen mediated by LOX and LHs have critical roles on the organization of collagen fibrils and serve as a template for bone mineralization as well as matrix formation.

## 3. Collagen Content in the Mandible Compared to the Long Bones

The biomechanical roles for collagen in bone are related to both the amount of collagen and its molecular stability and crosslinking. Bailey et al. [35] found that age-related decline in collagen content is nonlinearly correlated to the maximum stress at failure and to the modulus of elasticity in bone from human iliac crest. This does not mean that differences in collagen content are necessarily responsible for these changes but only that they are associated with them. Indeed, in other locations such as the femoral head and neck, no changes in collagen content were detected [36]. Other changes, such as the rate of turnover or degree of mineralization, might affect collagen content and the mechanical properties of bone independently. Despite such complex interactions, collagen content is representative of the status of bone matrix.

We have published the only study comparing the collagen content in the mandibular bone to long bones [37]. We calculated the collagen content in formalin-fixed human cortical bones from 44 cadavers at 3 different sites: the mental region of mandible, the mesial neck of humerus, and the mesial neck of femur. We found that the collagen content was significantly greater in the mandible (165.2 *μ*g/mg of dried bone weight) than in the humerus (146.4 *μ*g/mg) and femur (139.5 *μ*g/mg). Silva et al. [15] compared the bone matrix and mechanical properties of the femur in male SAMP6 mutant mice, a murine model of senile osteoporosis mice, to control SAMR1 mice, a senescence-resistant mutant mouse. They found that both demineralized and intact bones had greater reductions in mechanical strength in SAMP6 mice compared to the SAMR1 mice and that the cortical diaphysis also had a smaller amount of collagen in the SAMP6 mutants (97 *μ*g/mg of dried bone weight at 4 months of age, 106 *μ*g/mg at 12 months of age) versus SAMR1 mutants (113 *μ*g/mg at 4 months of age, 119 *μ*g/mg at 12 months of age). We [14] also investigated the cortical mandibles in males from the same mutant mouse strains at 6 months of age and obtained data similar to that by Silva et al. [15]. The mandible possessed a smaller amount of collagen in SAMP6 (126.4 *μ*g/mg of dried bone weight) than in SAMR1 (149.5 *μ*g/mg). Collagen fibers were also thinner in the SAMP6 mice (35.77 nm) than in the SAMR1 mice (43.71 nm). Despite the difference of age analysis between the two studies, mandibular bone displayed a greater amount of collagen compared to the femoral bone in the two mouse models.

The calvaria, which has the same origin as the jawbones [2], has similar tendency in collagen content. The research group of van den Bos et al. [38] investigated matrix composition of calvaria and long bones (femur, tibia, ulna, and radius) in female mice at 6 months of age and showed that the calvaria (302 *μ*g/mg of dried bone weight) had a greater amount of collagen than the long bones (211 *μ*g/mg).

As the collagen content mentioned above is calculated based on the value of hydroxyproline, it is mostly type I with trace amounts of types III [16] and V [17]. The comparison of type III or type V collagen content between the mandible and long bones has not been performed. Type III collagen is codistributed with type I collagen and is rich in Sharpey's fibers at the periodontal ligament and periosteum, penetrating to the bone [39]. In the mandible, Sharpey's fibers at the periodontal ligament across the entire thickness of alveolar wall and the fibers at the periosteum also penetrate to the cortical bone but become fewer, fragmented, superficial, and shortened with age [40]. In the femur, it has been revealed that the periosteal Sharpey fibers are rich at the trochanter and neck regions and penetrate to the cortical bone but decrease the density to the distal portion [41]. Exercise increases the density [42], while ovariectomy decreases it [41]. Type V collagen assembles with type I collagen into heterotypic fibrils [43]. The helical domain of type V collagen is buried within the fibril and type I collagen molecules are present along the fibril surface. The retained N-terminal domains of type V collagen are exposed at the surface and alter accretion of collagen molecules onto fibrils and then lateral growth. In bone, type V collagen does not show so specific distribution that type III collagen does. It shows a weak immunohistochemical staining in bone matrix [44], being preferably at the pericellular area but not in Sharpey's fibers [45].

The collagen content is present at similar levels between cortical and trabecular bones and between male and female [46]. Therefore, it is thought that the mandibular bone matrix including the trabecular bone is rich in collagen. The physiological basis of high collagen content in the mandible is unclear. One possible explanation is a higher rate of collagen turnover in the mandibular bone [1]. It therefore has properties of immature bone, which presumably have a low degree of mineralization resulting in a greater amount of collagen. Collagen fibrils contribute to bone flexibility, while mineral increases bone stiffness [13]. As a result, the mandible is more flexible than the long bones. This mechanical property leaves the bone well adapted to the constant, multidirectional forces associated with chewing and speaking. Another possible explanation for the high abundance of mandibular collagen is the relatively low amount of noncollagenous proteins. Though the mineral and collagen contents usually show a negative correlation, a decrease of collagen is occasionally compensated by an increase of noncollagenous proteins [47]. If the mandible has a smaller amount of noncollagenous proteins, it would then have a greater proportion of collagen. We will now further discuss the characteristics of the posttranslational modifications of collagen in the mandible.

## 4. Posttranslational Modifications of Collagen in the Mandible Compared to the Long Bones

There is no published data that directly compares collagen crosslinking between the mandible and long bones. In a previous study [14], we compared collagen crosslinking in the mandible of osteoporotic SAMP6 mice and control SAMR1 mice. As shown in Table 1, compared to SAMR1 mice, SAMP6 mice showed a smaller amount of the most abundant immature crosslink, deH-DHLNL (1.16 moles/mole of collagen in SAMP6, 1.30 moles/mole in SAMR1) but the same amount of the major mature crosslink, Pyr (0.34 moles/mole). The two mouse models exhibited the same level of the other measurable crosslinks: the immature crosslink, deH-HLNL (0.12 moles/mole), and the mature crosslink, d-Pyr (0.02 moles/mole). SAMP6 showed a smaller amount of total crosslinks (1.64 moles/mole in SAMP6, 1.79 moles/mole in SAMR1) due to a decrease in the most abundant crosslink, deH-DHLNL, and a higher rate of collagen maturation (Pyr/deH-DHLNL, 0.29 in SAMP6, 0.25 in SAMR1).

Silva et al. [15] also reported the amount of mature crosslinks, Pyr and d-Pyr, in the femur of the same mouse models at 4 months and 12 months of age. In this study, Pyr showed similar levels between SAMP6 and SAMR1 mice at each age, but the values were greater in the older animals as did d-Pyr crosslinks (Table 1).

Because we [14] used the same mouse models as Silva et al. [15], the data quantifying mature crosslinks can be compared between the mandible and the femur. Although the mandible was tested at an older age than the femur (6 months versus 4 months of age, resp.), the amount of Pyr in the mandible versus the femur of SAMP6 and SAMR1 mice was 52% and 55% smaller, respectively. Similarly, the amount of d-Pyr is 67% smaller in SAMP6 mice and 71% smaller in SAMR1 mice. Given that aging increases the amount of mature crosslinks, it is likely that the mandible has a lower amount of mature crosslinks. Although we cannot compare immature crosslinks between mandible and long bones directly, an abundant quantity of deH-DHLNL, indeed, exists in mandible (Table 1). Thus, we speculate that mandible has a higher rate of immature bone collagen. The high rate of the immature crosslinks allows for easy degradation of the matrix [25] and possibly a lower degree of mineralization [18]. These properties are associated with a high rate of bone turnover.

As for another important posttranslational modification of collagen, Lys hydroxylation, we previously published a comparative study of the mandible, the humerus, and the femur in formalin-fixed cadavers [37]. The extent of Lys hydroxylation (Hyl/Lys + Hyl) was lower in the mandible (11.9%) than in the humerus (14.8%) and the femur (13.7%). However, this data has some caveats. Because formalin crosslinks Lys and Hyl residues and causes formation of their derivatives, the value of Lys and Hyl quantified by amino acid analysis may have diminished during fixation [48]. We also note that Lys hydroxylation varies across different regions of the same bone [49]. The lower Lys hydroxylation in the mandible has a number of potential implications for mandible bone physiology. Bone in senile osteoporotic mice has impaired mechanical function correlated with increased Lys hydroxylation and decreases in collagen amount [14, 15] and in thickness of collagen fibrils [14]. These data are in accordance with findings from other studies showing that overhydroxylation of Lys leads to impairment of collagen fibril formation and bone matrix organization [27, 50]. The lower Lys hydroxylation in the mandible connotes thicker collagen fibrils, which accord with the greater amount of collagen. However, the collagen fibrillogenesis is complex and elaborate by not only its posttranslational modifications but also other factors such as small leucine-rich proteoglycans [51], minor collagens, fibronectin, and integrins [52]. The collagen fibril thickness needs to be investigated.

**Figure 3:**
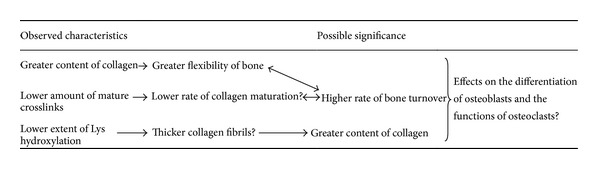
The collagen characteristics of the mandibular bone compared to the long bones and their possible significance.

As shown in Figure 3, the differences in collagen characteristic between the normal and osteoporotic bones are similar to those between the mandibular and long bones. The greater amount of collagen, lower rate of cross-link maturation, and lower extent of Lys hydroxylation in the mandible are suggestive of the higher rate of bone turnover and greater bone flexibility. In fact, high turnover and greater flexibility in mandibular bone are likely necessary to endure the constant and multidirectional forces of routine activities like chewing and speaking. Notably, the force placed on the mandible during mastication is almost twice as intense to the force generated during walking [53, 54]. Further investigation will test these hypotheses.

## 5. Relevance to Clinical Dentistry

It is important for dentists and dental researchers to understand the specific features of jaw physiology and its impact on the matrix of the jawbones. The jaw has interesting properties related to its function and age-related change in bone volume. Aging is associated with atrophy but not fracture in the jaw. The most plausible explanation is that the jaw undergoes frequent exercise but is not weight bearing. Unfortunately, collagen in bones of the jaw is given less attention in the literature than other skeletal bones. By focusing on collagen, this review addressed limited but essential aspects of jawbone remodeling and biochemical properties.

Bone is a dynamic tissue that is constantly remodeled by osteoblasts and osteoclasts differentiated from bone marrow mesenchymal or stromal cells. These cells not only produce new bone but are also regulated by the bone matrix. Collagen crosslinking likely influences osteoblastic differentiation. For instance, lathyrogens inhibit the production of deH-DHLNL and Pyr in bone matrix produced by osteoblasts. The disturbed matrix, in turn, influences the osteoblasts by inducing upregulation of type I collagen mRNA and downregulation of osteocalcin mRNA. This suggests that optimal crosslinking accelerates osteoblastic differentiation [55].

Everts et al. hypothesize that the differences in the amount of mature crosslinks and the concomitant degradability of collagen may offer an explanation for the functional heterogeneity of proteases necessary for proper resorption of bone by osteoclasts [38, 56, 57]. Calvarial bone has a greater amount of collagen, a smaller amount of mature crosslinks, and more degradability of collagen with pepsin, cathepsin K, or matrix metalloproteinase (MMP)-2 compared with long bones [38]. The long bone osteoclasts primarily use cysteine proteinases (e.g., cathepsin K) to degrade the mature cross-linked matrix, whereas the calvarial osteoclasts resorb immature cross-linked matrix through MMPs as well [56, 57]. The mandible appears similar to calvaria in total amount of collagen and mature crosslinks. Although the proteinases used for resorption in the mandible are not clear, the osteoclasts do exhibit a number of different properties from those in the long bones [58–60].

Here we have reviewed functionally important and unique characteristics of collagen in the mandible compared to the long bones, including a greater amount of collagen, a smaller amount of mature crosslinks, and a smaller extent of Lys hydroxylation (Figure 3). These properties necessitate a high rate of collagen turnover to meet the mechanical needs of the mandible and the distinct interactions of the mandibular matrix with osteoblasts, osteoclasts, and their precursors.

To the best of our knowledge, there are no reports that characterize maxillary collagen biochemistry. Though they share a common developmental origin, the maxilla exhibits a number of key differences when compared to the mandible, such as absence of the Meckel cartilage during the developmental process [61], more porosity of bone, and a lower rate of bone turnover [1]. Clinical observations demonstrate that the maxilla has lower density and stiffness at the site of dental implant insertion [62]. Indeed, dentists often observe that a smaller force is required for tooth extraction. Though dental implant placed into the maxilla shows a sufficiently high success rate, it is nevertheless lower compared to that for the mandible [63]. In contrast, implant survival rate and changes in the marginal bone level are not associated with bone density or stiffness [62].

These clinical phenomena cannot be explained by differences in the anatomical structure or stiffness of bone alone. Because collagen also regulates cellular activities and bone remodeling, it is likely critical for anchorage and long-term maintenance of teeth and dental implants as well as the preservation of alveolar bones. Further investigation of jawbone collagen's unique biochemical properties, relationship to the matrix, and cellular interactions is needed for dentists to develop better clinical practices and introduce new technologies based on sound scientific evidence.

## Figures and Tables

**Table 1:** Collagen cross-links of the mandible and the femur from SAM mice.

		Mouse	Mandible (*n* = 6)	Femur (*n* = 10)	
			6 months	4 months	12 months
Immature crosslinks	deH-DHLNL	SAMR1	1.30 ± 0.01	—	—
		SAMP6	1.16 ± 0.06	—	—
	deH-HLNL	SAMR1	0.12 ± 0.01	—	—
		SAMP6	0.12 ± 0.02	—	—

Mature crosslinks	Pyr	SAMR1	0.34 ± 0.02	0.62 ± 0.03	0.80 ± 0.02
		SAMP6	0.34 ± 0.02	0.65 ± 0.02	0.84 ± 0.04
	d-Pyr	SAMR1	0.02 ± 0.01	0.028 ± 0.011	0.052 ± 0.007
		SAMP6	0.02 ± 0.00	0.030 ± 0.011	0.048 ± 0.009

Values show mean ± SD (mol/mol collagen). The data of the mandible and the femur was reported by Tokutomi et al. [14] and Silva et al. [15], respectively.
